# Supplementary material for: Obstructive sleep apnea mediates genetic risk of Diabetes Mellitus in Hispanic and Latino communities
Source: Commun Med (Lond). 2025 Sep 25;5:398. doi: 10.1038/s43856-025-01107-y (PMC12462445; doi:10.1038/s43856-025-01107-y)
Supplement: Supplementary file 2 — Supplementary Information [file 43856_2025_1107_MOESM2_ESM.pdf]

Obstructive sleep apnea mediates genetic risk of Diabetes Mellitus in the Hispanic and Latino Communities

Supplementary Information

Yana Hrytsenko, Brian W. Spitzer, Heming Wang, Suzanne M. Bertisch, Kent Taylor, Olga Garcia-Bedoya, Alberto R. Ramos, Martha L. Daviglus, Linda C. Gallo, Carmen Isasi, Jianwen Cai, Qibin Qi, Carmela Alcantara, Susan Redline, Tamar Sofer

Table of Contents

Supplementary Figures **3**

Supplementary Figure 1: Distribution of multi-ancestry T2D-PRSs by DM category between visit 1 and 2 to the clinic. 3

Supplementary Figure 2: Association of multi-ancestry T2D-PRSs with DM and incident DM stratified by self-reported ancestral background. 4

Supplementary Figure 3: T2D-PRSs associations with DM stratified by OSA categories. 5

Supplementary Figure 4: Interaction between multi-ancestry T2D-PRSs and OSA phenotypes in association with incident DM. 6

Supplementary Figure 5: Comparison of model performance for T2D-PRSs association with DM and incident DM using covariates and covariates plus T2D-PRS model. 7

Supplementary Figure 6: Association of multi-ancestry T2D-PRSs with poor sleep health. 8

Supplementary Figure 7: Association of multi-ancestry with T2D-PRSs with OSA comparing adjustment for BMI to WHR. 9

Supplementary Figure 8: Mediation effect of OSA on associations of mgbPRSsum with DM. 10

Supplementary Figure 9: Mediation effect of OSA on associations of BMIadjT2D-PRS with DM. 11

Supplementary Figure 10: Estimated causal effects of OSA on T2D. 12

Supplementary Figure 11: Association of OSA-PRS with OSA at baseline 12

Supplementary Figure 12: Association of OSA-PRS with DM at baseline 13

Supplementary Note 1: Scanned anti-diabetic medications and self-reported antidiabetic medication use ***14***

Supplementary Tables ***15***

Supplementary Table 1: GWAS summary statistics used for T2D-PRS development. **15**

Supplementary Table 2: Characteristics of HCHS/SOL target population with no DM at baseline stratified by sleep phenotype categories. **16**

Supplementary Table 3: Characteristics of HCHS/SOL target population at baseline stratified by sleep phenotype categories. **17**

Supplementary Table 4: Characteristics of MGB dataset stratified by T2D status. **18**

Supplementary Table 5: Characteristics of HCHS/SOL target population stratified by self-reported Hispanic background. **19**

Supplementary Table 6: Associations of T2D PRS with OSA in analyses adjusted for WHR instead of BMI **20**

Supplementary Table 7: Estimated associations between T2D PRSs and moderate-to-severe OSA when adjusting for potential confounders of the T2D-OSA association **21**

Supplementary Table 8: Estimated associations between T2D PRSs and mild-to-severe OSA when adjusting for potential confounders of the T2D-OSA association **22**

Supplementary Table 9: Results from bidirectional multivariate MR (MVMR) analysis of T2D and OSA adjusted for BMI **23**

Supplementary References ***24***

Supplementary Figures

Supplementary Figure 1: Distribution of multi-ancestry T2D-PRSs by DM category between visit 1 and 2 to the clinic.

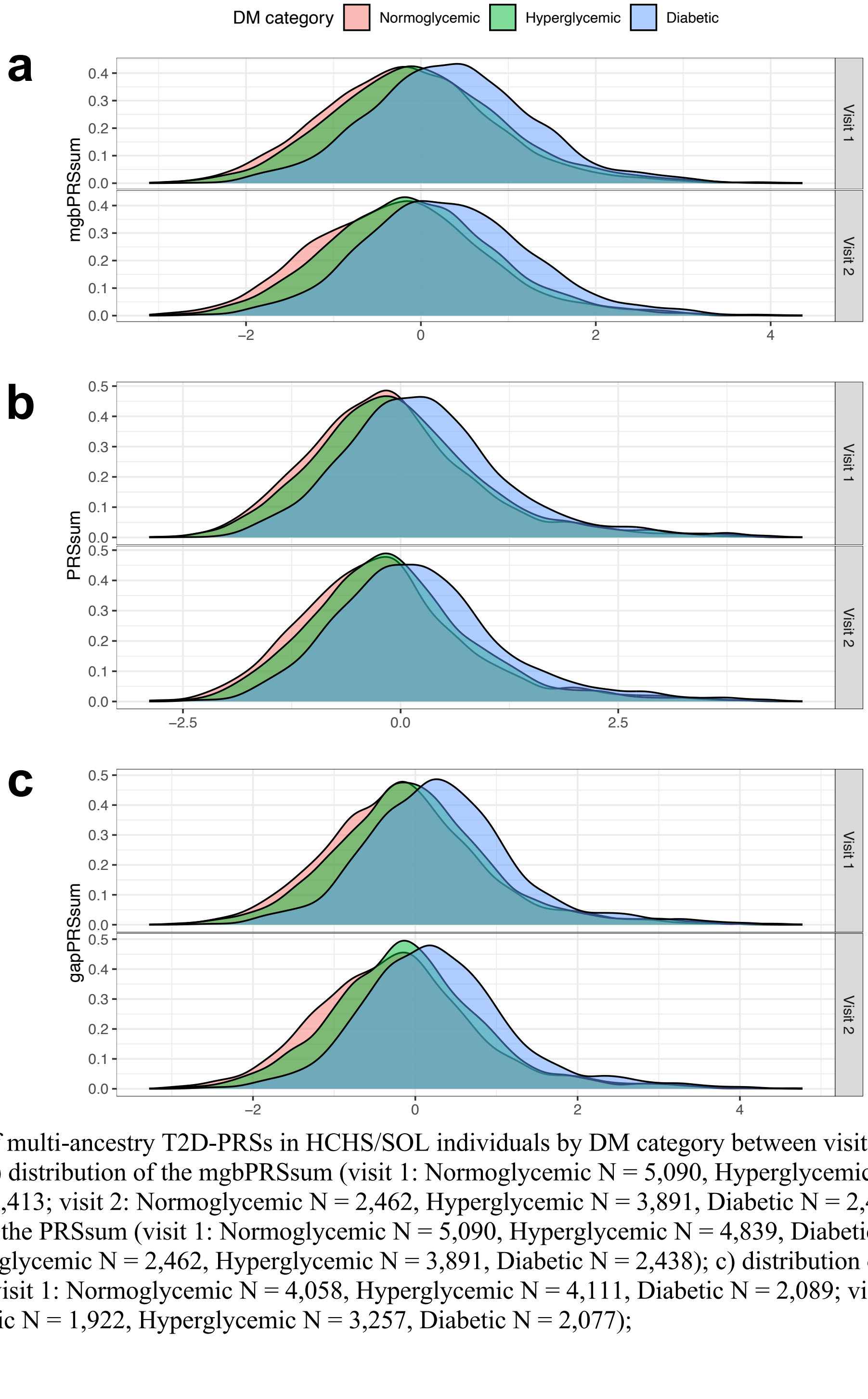

Distribution of multi-ancestry T2D-PRSs in HCHS/SOL individuals by DM category between visit 1 and 2 to the clinic. Panel a) distribution of the mgbPRSsum (visit 1: Normoglycemic N = 5,090, Hyperglycemic N = 4,839, Diabetic N = 2,413; visit 2: Normoglycemic N = 2,462, Hyperglycemic N = 3,891, Diabetic N = 2,438); b) distribution of the PRSsum (visit 1: Normoglycemic N = 5,090, Hyperglycemic N = 4,839, Diabetic N = 2,413; visit 2: Normoglycemic N = 2,462, Hyperglycemic N = 3,891, Diabetic N = 2,438); c) distribution of the gapPRSsum (visit 1: Normoglycemic N = 4,058, Hyperglycemic N = 4,111, Diabetic N = 2,089; visit 2: Normoglycemic N = 1,922, Hyperglycemic N = 3,257, Diabetic N = 2,077);

Supplementary Figure 2: Association of multi-ancestry T2D-PRSs with DM and incident DM stratified by self-reported ancestral background.

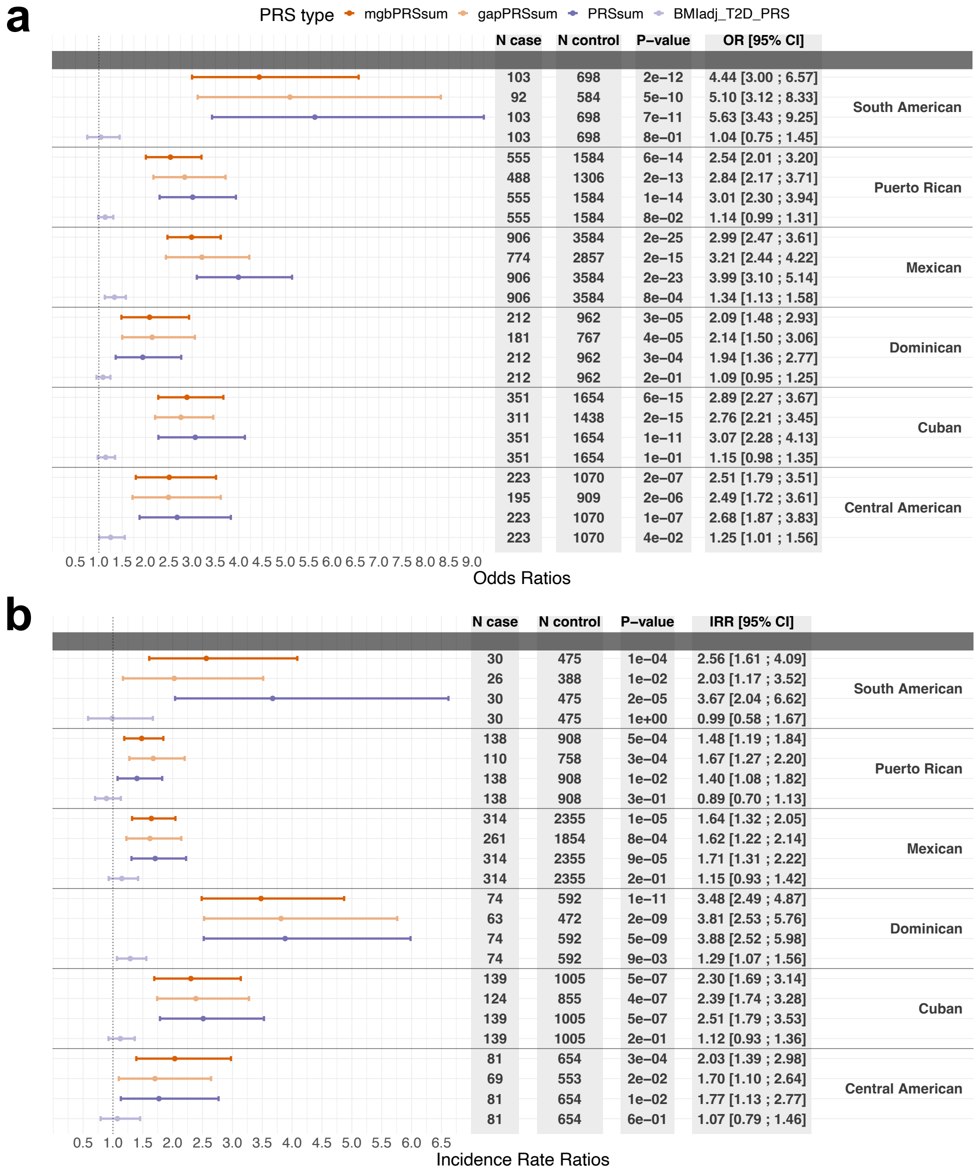

Panel a) Estimated OR per 1 SD increase in T2D-PRSs in association with DM at baseline in HCHS/SOL individuals with DM status at baseline b) Estimated IRR of T2D-PRS in association with incident DM in individuals free of DM at baseline. Results are stratified by Hispanic/Latino background. PRSs were standardized in the full dataset, rather than by a specific sample used in a given analysis. Throughout, error bars represent 95% confidence intervals.

DM: Diabetes Mellitus; OR: odds ratio; IRR: incidence rate ratios; PRS: polygenic risk score; SD: standard devision.

Supplementary Figure 3: T2D-PRSs associations with DM stratified by OSA categories.

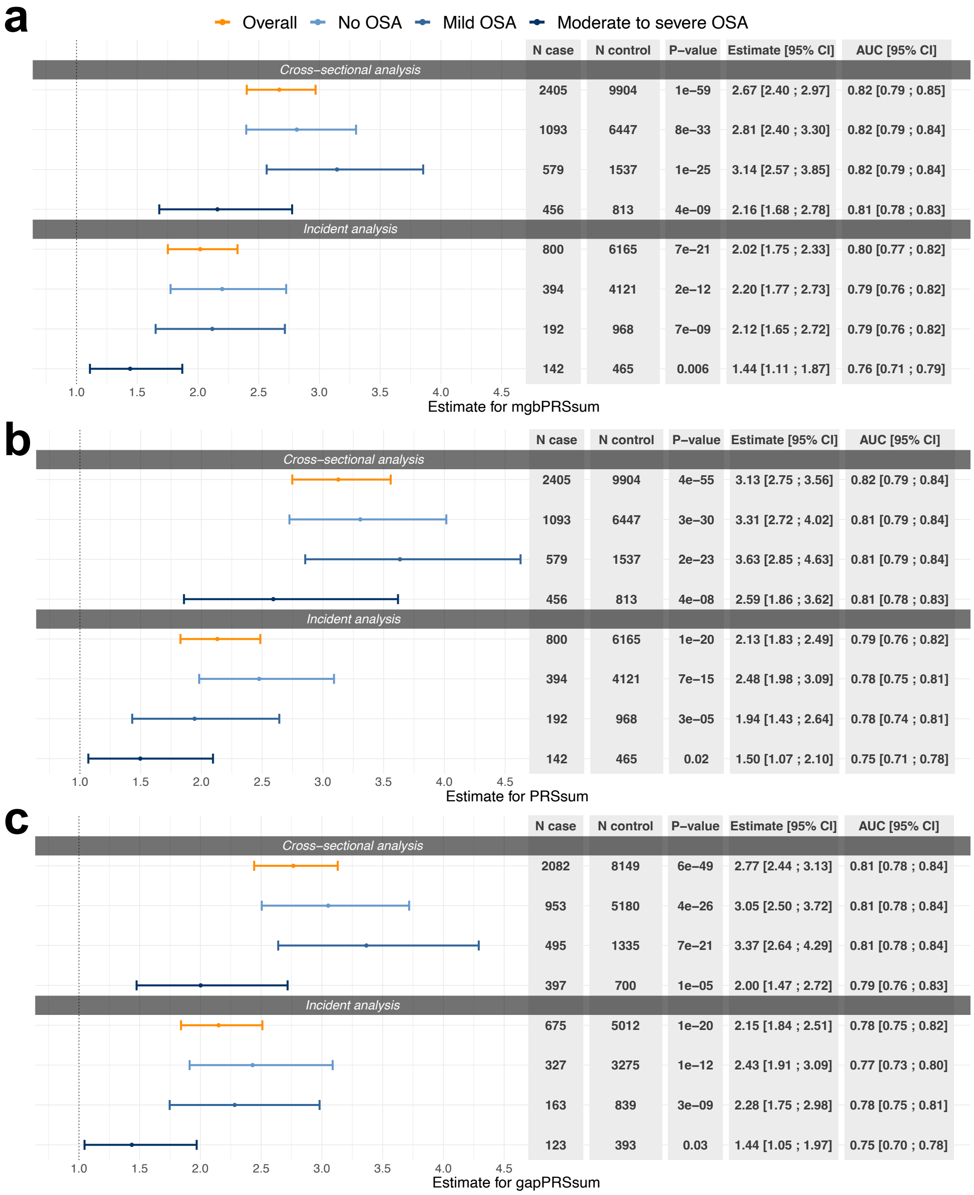

Panel **a)** Association of 1 SD increase in T2D-PRSs with DM and incident DM in HCHS/SOL individuals stratified by OSA severity levels and overall dataset. **b)** Association of 1 SD increase in PRSsum T2D-PRSs with DM and incident DM in HCHS/SOL individuals stratified by OSA categories and overall dataset. **c)** Association of 1 SD increase in gapPRSsum T2D-PRSs with DM and incident DM in HCHS/SOL individuals stratified by OSA categories and overall dataset. PRSs were standardized in the full dataset, rather than by a specific sample used in a given analysis. Throughout, error bars represent 95% confidence intervals.
All models were adjusted for age, sex, BMI, study center and 5 genetic PCs.
OR: odds ratios; IRR: incidence rate ratios; AUC: Area Under the ROC (receiver operating characteristic) Curve; T2D: type 2 diabetes; PRSs: polygenic risk scores; DM: diabetes mellitus; HCHS/SOL: Hispanic Community Health Study/Study of Latinos; EDS: excessive daytime sleepiness; OSA: obstructive sleep apnea; SD: standard deviation.

Supplementary Figure 4: Interaction between multi-ancestry T2D-PRSs and OSA phenotypes in association with incident DM.

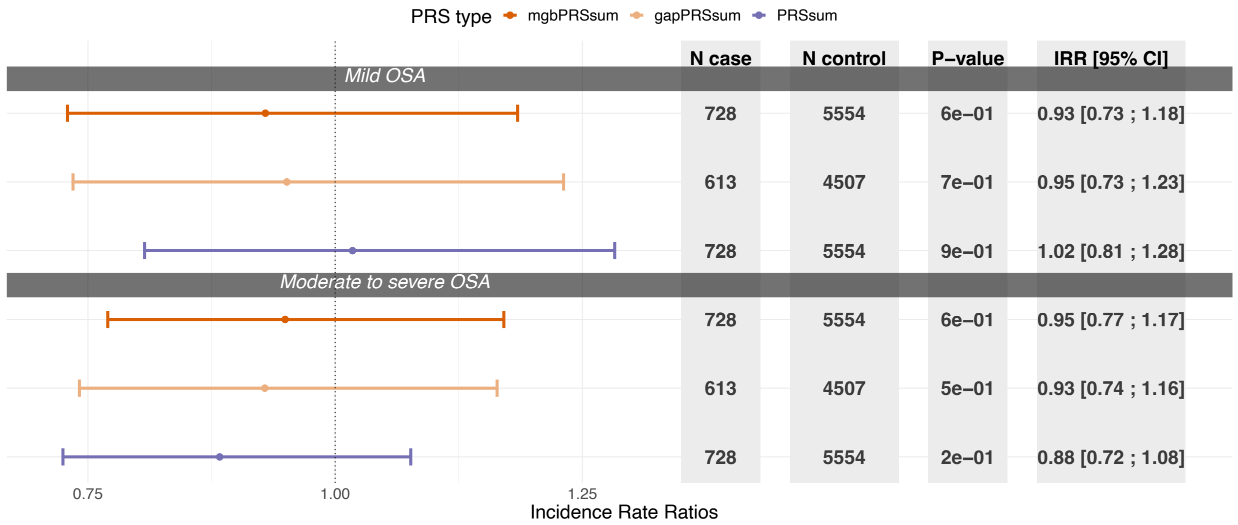

Estimated IRR of interaction between three types of T2D-PRSs and OSA in association with incident DM among normoglycemic and hyperglycemic individuals at baseline. Results are stratified by OSA severity categories. IRRs were estimates per 1 SD of the PRSs. PRSs were standardized in the full dataset, rather than by a specific sample used in a given analysis. Throughout, error bars represent 95% confidence intervals.
DM: Diabetes Mellitus; IRR: incidence rate ratio; OSA: obstructive sleep apnea; PRS: polygenic risk score; SD: standard deviation.

Supplementary Figure 5: Comparison of model performance for T2D-PRSs association with DM and incident DM using covariates and covariates plus T2D-PRS model.

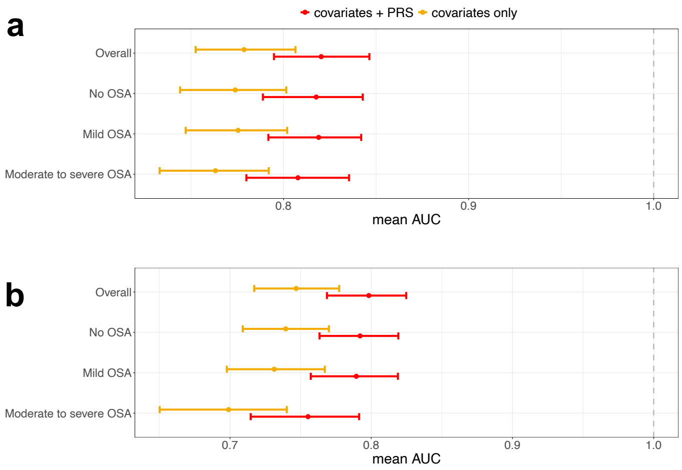

Panel **a)** Mean AUC of the "covariates model" in comparison to the performance of the "covariates plus T2D-PRS model" in estimating association between T2D-PRS and DM at baseline (testing set: overall N = 1218, no OSA N = 1226, mild OSA N = 1254, moderate to severe OSA N = 1229). **b)** Mean AUC of the "covariates model" in comparison to the performance of the "covariates plus T2D-PRS model" in estimating association between T2D-PRS and incident DM (testing set: overall N = 877, no OSA N = 875, mild OSA N = 877, moderate to severe OSA N = 880). Throughout, error bars represent 95% confidence intervals.
AUC: area under the ROC curve; OSA: obstructive sleep apnea.

Supplementary Figure 6: Association of multi-ancestry T2D-PRSs with poor sleep health.

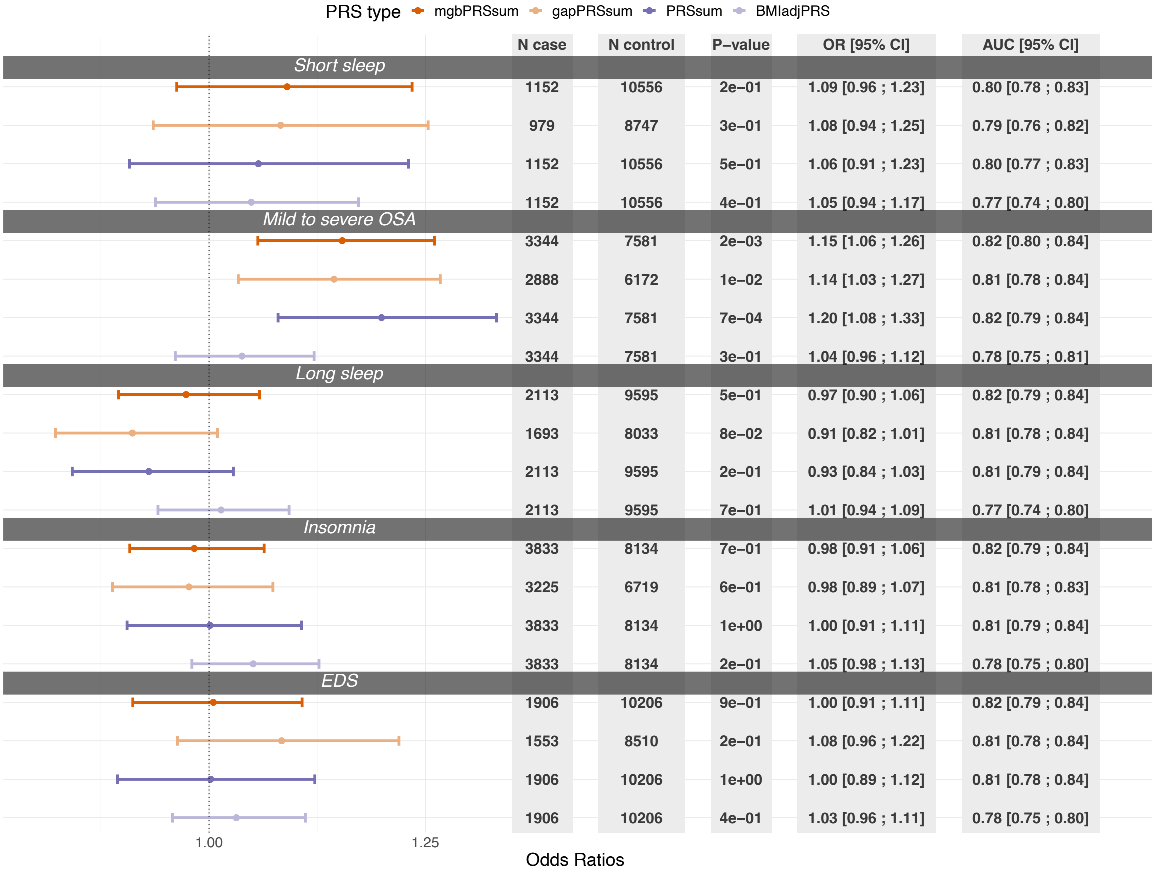

Category	PRS type	N case	N control	P–value	OR [95% CI]	AUC [95% CI]
Short sleep	mgbPRSsum	1152	10556	2e–01	1.09 [0.96 ; 1.23]	0.80 [0.78 ; 0.83]
	gapPRSsum	979	8747	3e–01	1.08 [0.94 ; 1.25]	0.79 [0.76 ; 0.82]
	PRSsum	1152	10556	5e–01	1.06 [0.91 ; 1.23]	0.80 [0.77 ; 0.83]
	BMIadjPRS	1152	10556	4e–01	1.05 [0.94 ; 1.17]	0.77 [0.74 ; 0.80]
Mild to severe OSA	mgbPRSsum	3344	7581	2e–03	1.15 [1.06 ; 1.26]	0.82 [0.80 ; 0.84]
	gapPRSsum	2888	6172	1e–02	1.14 [1.03 ; 1.27]	0.81 [0.78 ; 0.84]
	PRSsum	3344	7581	7e–04	1.20 [1.08 ; 1.33]	0.82 [0.79 ; 0.84]
	BMIadjPRS	3344	7581	3e–01	1.04 [0.96 ; 1.12]	0.78 [0.75 ; 0.81]
Long sleep	mgbPRSsum	2113	9595	5e–01	0.97 [0.90 ; 1.06]	0.82 [0.79 ; 0.84]
	gapPRSsum	1693	8033	8e–02	0.91 [0.82 ; 1.01]	0.81 [0.78 ; 0.84]
	PRSsum	2113	9595	2e–01	0.93 [0.84 ; 1.03]	0.81 [0.79 ; 0.84]
	BMIadjPRS	2113	9595	7e–01	1.01 [0.94 ; 1.09]	0.77 [0.74 ; 0.80]
Insomnia	mgbPRSsum	3833	8134	7e–01	0.98 [0.91 ; 1.06]	0.82 [0.79 ; 0.84]
	gapPRSsum	3225	6719	6e–01	0.98 [0.89 ; 1.07]	0.81 [0.78 ; 0.83]
	PRSsum	3833	8134	1e+00	1.00 [0.91 ; 1.11]	0.81 [0.79 ; 0.84]
	BMIadjPRS	3833	8134	2e–01	1.05 [0.98 ; 1.13]	0.78 [0.75 ; 0.80]
EDS	mgbPRSsum	1906	10206	9e–01	1.00 [0.91 ; 1.11]	0.82 [0.79 ; 0.84]
	gapPRSsum	1553	8510	2e–01	1.08 [0.96 ; 1.22]	0.81 [0.78 ; 0.84]
	PRSsum	1906	10206	1e+00	1.00 [0.89 ; 1.12]	0.81 [0.78 ; 0.84]
	BMIadjPRS	1906	10206	4e–01	1.03 [0.96 ; 1.11]	0.78 [0.75 ; 0.80]

Estimated OR per 1 SD increase in T2D-PRS in association with poor sleep health at baseline in HCHS/SOL individuals with DM status at baseline. Results are stratified by poor sleep health categories. PRSs were standardized in the full dataset, rather than by a specific sample used in a given analysis. Throughout, error bars represent 95% confidence intervals.

DM: Diabetes Mellitus; EDS: excessive daytime sleepiness; OR: odds ratio; OSA: obstructive sleep apnea; PRS: polygenic risk score; SD: standard deviation.

Supplementary Figure 7: Association of multi-ancestry with T2D-PRSs with OSA comparing adjustment for BMI to WHR.

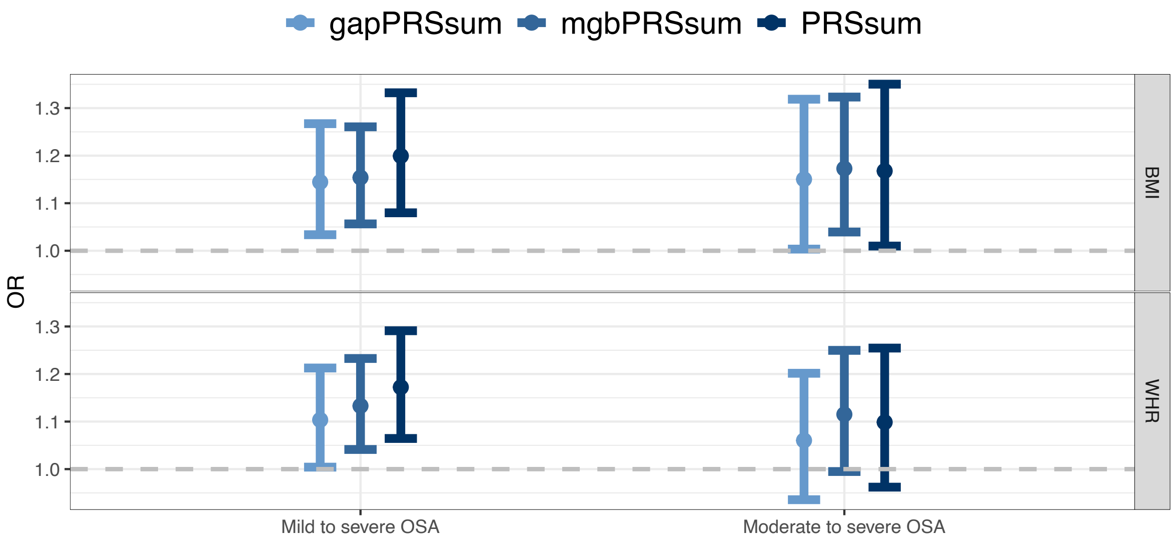

Comparison of estimated OR per 1 SD increase in T2D-PRS in association with OSA at baseline in HCHS/SOL individuals adjusting for BMI versus WHR. Results are stratified by OSA severity categories. Throughout, error bars represent 95% confidence intervals.

BMI: body mass index; WHR: waist to hip ratio; OR: odds ratio; OSA: obstructive sleep apnea. PRS: polygenic risk score; SD: standard deviation.

Supplementary Figure 8: Mediation effect of OSA on associations of mgbPRSsum with DM.

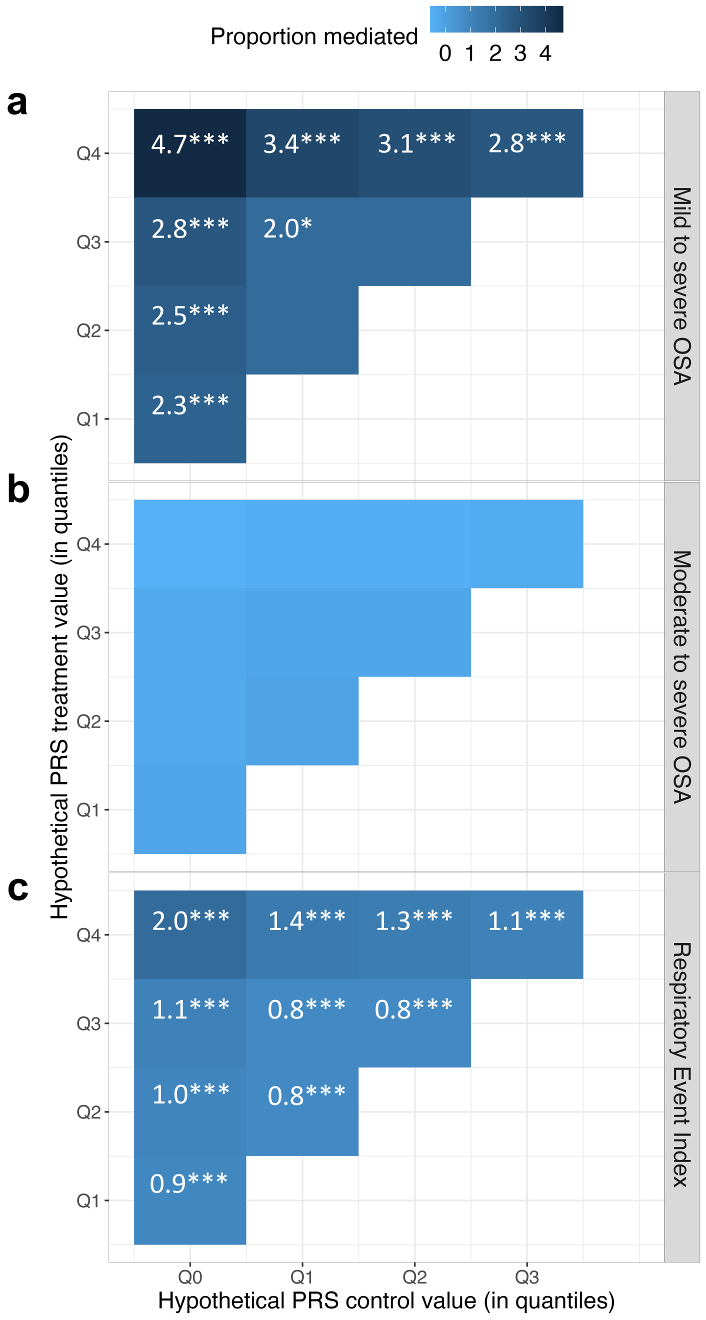

Panel a: Estimated proportion of mediation by the Moderate to severe OSA in the association between mgbPRSsum and incident DM in individuals who participated at the second visit to a clinic (N = 6,291). Panel b: Estimated proportion of mediation by the Moderate to severe OSA in the association between mgbPRSsum and incident DM in individuals who participated at the second visit to a clinic (N = 6,291). Panel c: Estimated proportion of mediation by REI in the association between mgbPRSsum and incident DM in individuals who participated at the second visit to a clinic (N = 6,291). Presented associations were estimated based on a change in values in the PRS, where values are set according to the sample distribution quantiles (Q0-Q4). Darker tile shades correspond to higher estimated values.

Significance codes: 0 >= '***' < 0.001 >= '**' < 0.01 >= '*' < 0.05 ' ' < 0.1

All models were adjusted for age, sex, BMI and 5 genetic PCs.

PRS: polygenic risk score; OSA: obstructive sleep apnea.

Supplementary Figure 9: Mediation effect of OSA on associations of BMIadjT2D-PRS with DM.

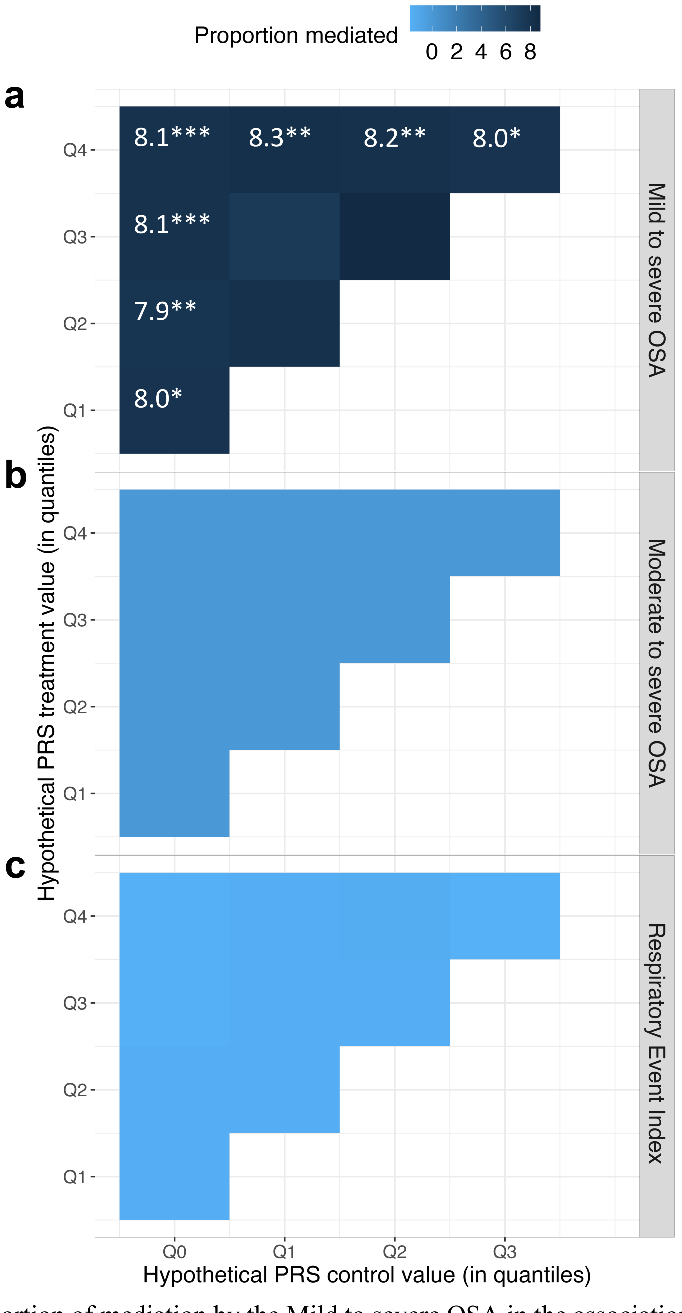

Panel a: Estimated proportion of mediation by the Mild to severe OSA in the association between BMIadjT2D-PRS and incident DM in individuals who participated at the second visit to a clinic (N = 6,291). Panel b: Estimated proportion of mediation by the Moderate to severe OSA in the association between BMIadjT2D-PRS and incident DM in individuals who participated at the second visit to a clinic (N = 6,291). Panel c: Estimated proportion of mediation by REI in the association between BMIadjT2D-PRS and incident DM in individuals who participated at the second visit to a clinic (N = 6,291). Presented associations were estimated based on a change in values in the PRS, where values are set according to the sample distribution quantiles (Q0-Q4). Darker tile shades correspond to higher estimated values.

Significance codes: 0 >= '***' < 0.001 >= '**' < 0.01 >= '*' < 0.05 ' ' < 0.1

All models were adjusted for age, sex, BMI and 5 genetic PCs.

PRS: polygenic risk score; OSA: obstructive sleep apnea.

Supplementary Figure 10: Estimated causal effects of OSA on T2D.

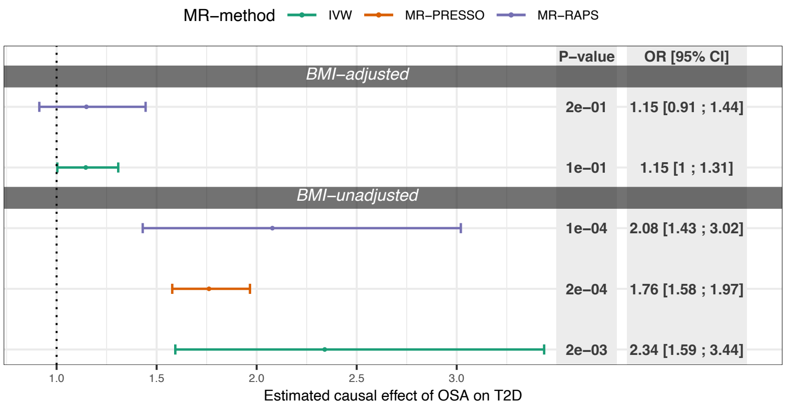

Figure: Estimated causal effect of OSA on T2D based on SNPs selected using p-value threshold < 10^{-7} in BMI-adjusted and BMI-unadjusted OSA GWASs. Throughout, error bars represent 95% confidence intervals. T2D: type 2 diabetes; OSA: obstructive sleep apnea; IVW: inverse variance weighted; BMI: body mass index; OR: odds ratios.

Supplementary Figure 11: Association of OSA-PRS with OSA at baseline

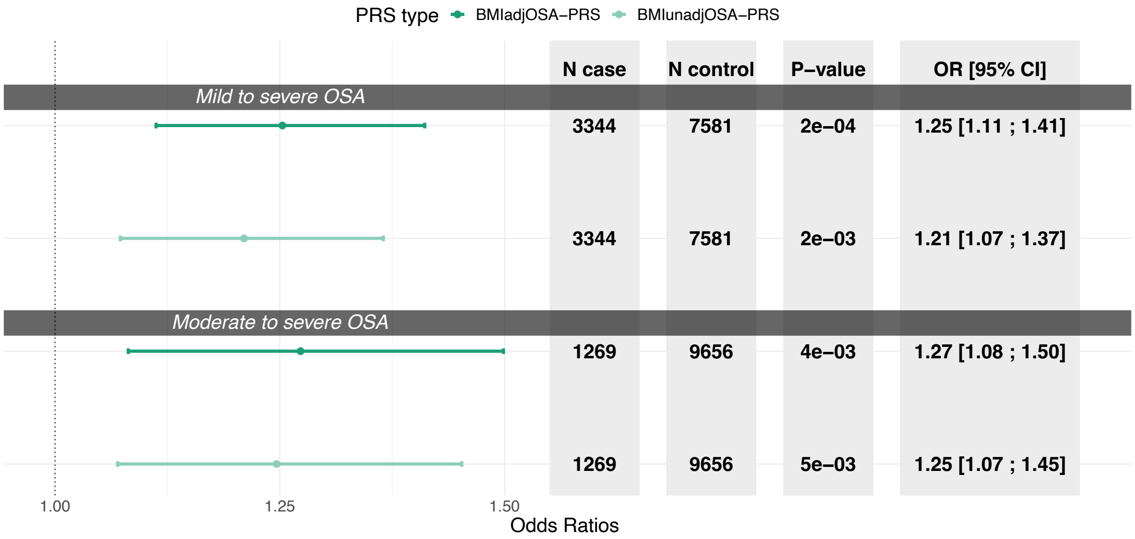

Association between OSA-PRSs (constructed using two OSA GWASs: BMI-adjusted and BMI-unadjusted) and OSA at baseline stratified by OSA severity categories: mild to severe OSA and moderate to severe. Associations were estimated as ORs per 1 SD increase in the PRS. PRSs were standardized in the full dataset, rather than by a specific sample used in a given analysis. Throughout, error bars represent 95% confidence intervals.

OSA: Obstructive Sleep Apnea; PRS: polygenic risk score; BMI: body mass index; GWAS: Genome Wide Association Study; OR: Odds Ratios; CI: confidence intervals; SD: standard deviation.

Supplementary Figure 12: Association of OSA-PRS with DM at baseline

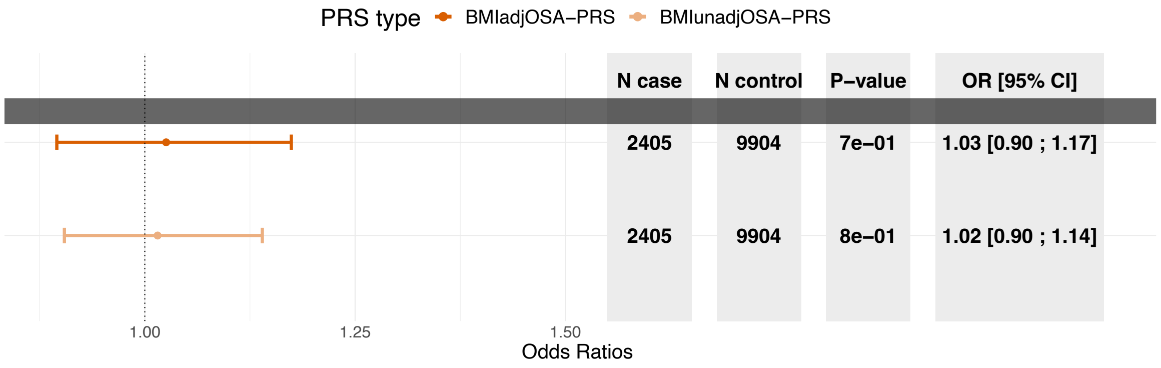

Association between OSA-PRSs (constructed using two OSA GWASs: BMI-adjusted and BMI-unadjusted) and DM at baseline in the overall dataset (N = 12,309). Associations were estimated as ORs per 1 SD increase in the PRS. PRSs were standardized in the full dataset, rather than by a specific sample used in a given analysis. Throughout, error bars represent 95% confidence intervals.

OSA: Obstructive Sleep Apnea; PRS: polygenic risk score; OR: Odds Ratios; CI: confidence intervals; SD: standard deviation.

Supplementary Note 1: Scanned anti-diabetic medications and self-reported antidiabetic medication use

Scanned anti-diabetic medications included: “antidiabetics” , “insulin” , “mixed insulin”, “beef insulin”, “pork insulin”, “human insulin”, “antidiabetic - amylin analogs”, “sulfonylureas”, “sulfoylurea combinations”, “antidiabetic - amino acid derivatives”, “antidiabetic-d-phenylalanine derivatives”, “biguanides”, “meglitinide analogues”, “meglitinide analogues”, “diabetic other”, “diabetic other - combinations”, “aldose reductase inhibitors”, “alpha-glucosidase inhibitors”, “insulin sensitizing agents”, “thiazolidinediones”, “antidiabetic combinations”, “sulfonylurea-biguanide combinations”. Self-reported antidiabetic medication use was determined by the “Yes” answer to following question: “Were any of the medications you took during the last 4 weeks for high blood sugar or diabetes?”.

Supplementary Tables

Supplementary Table 1: GWAS summary statistics used for T2D-PRS development.

GWAS name	Reference	Trait	Population
DIAGRAM	PMID: 35551307 [2]	T2D	180,834 T2D cases and 1,159,055 controls (effective sample size 492,191) across five ancestry groups: European ancestry (51.1% of the total effective sample size); East Asian ancestry (28.4%); South Asian ancestry (8.3%); African ancestry, including recently admixed African American populations (6.6%); and Hispanic individuals with recent admixture of American, African, and European ancestry (5.6%).
MVP	PMID: 32541925 [3]	T2D	228,499 cases and 1,178,783 controls encompassing five ancestral groups (Europeans, African Americans, Hispanics, South Asians and East Asians). MVP participants (n = 273,409) comprised predominantly male subjects (91.6%) and were classified as Europeans (72.1%), African Americans (19.5%), Hispanics (7.5%), and Asians (0.9%).

The table provides T2D GWAS source, study population as reported by the manuscript reporting the GWAS, and number of participants used to generate summary statistics. Bold font represent column names.

Supplementary Table 2: Characteristics of HCHS/SOL target population with no DM at baseline stratified by sleep phenotype categories.

**Characteristic**	**All**	**Healthy sleep**	**Insomnia**	**Short sleep**	**Long sleep**	**EDS**	**No OSA**	**Mild OSA**	**Moderate to severe OSA**
N	9,929	3,225	3,844	1,158	2,121	1,909	7,563	2,122	1,270
Gender N (%)									
Female	5,795 (50.5)	1,686 (50.4)	1,957 (59.5)	479 (46.7)	1,062 (54.9)	840 (50.4)	4,091 (54.7)	768 (39.6)	296 (29.2)
Male	4,134 (49.5)	1,126 (49.6)	972 (40.5)	410 (53.3)	619 (45.1)	622 (49.6)	2,374 (45.3)	772 (60.4)	518 (70.8)
Age									
Mean (SD)	39.23 (14.29)	36.49 (12.96)	42.19 (14.05)	40.84 (14.15)	34.97 (14.56)	40.52 (14.66)	36.40 (13.35)	48.26 (12.58)	50.21 (13.14)
BMI									
Mean (SD)	28.92 (5.89)	27.83 (5.09)	29.59 (6.37)	29.93 (6.02)	28.18 (5.92)	29.58 (6.12)	28.04 (5.56)	31.12 (5.81)	33.31 (5.92)
DM status at visit 1 N (%)									
Normoglycemic	5,090 (56.9)	1,651 (57.5)	1,387 (43.4)	416 (42.4)	959 (54.6)	722 (44.3)	3,742 (56.8)	544 (28.8)	216 (18.3)
Hyperglycemic	4,839 (36.6)	1,161 (33.8)	1,542 (37.2)	473 (41.2)	722 (30.2)	740 (38.2)	2,723 (33.1)	996 (46.8)	598 (47.1)
DM status at visit 2 N (%)									
Normoglycemic	2,437 (26.3)	821 (30.1)	671 (23.2)	203 (22.6)	434 (24.3)	339 (25.7)	1,845 (29.8)	251 (17.7)	95 (11.3)
Hyperglycemic	3,735 (32.3)	995 (30.0)	1,173 (35.7)	356 (35.1)	567 (23.3)	605 (37.7)	2,283 (29.5)	719 (43.0)	369 (45.4)
Diabetic	803 (6.1)	162 (4.1)	282 (7.4)	68 (6.0)	403 (13.4)	149 (8.1)	394 (4.4)	192 (11.2)	143 (14.4)
Missing DM status	2,954 (35.4)	834 (35.8)	803 (33.7)	262 (36.3)	717 (39.0)	369 (28.4)	1,943 (36.3)	378 (28.0)	207 (28.8)

Bold font represent column names.

Supplementary Table 3: Characteristics of HCHS/SOL target population at baseline stratified by sleep phenotype categories.

**Characteristic**	**All**	**Healthy sleep**	**Insomnia**	**Short sleep**	**Long sleep**	**EDS**	**No OSA**	**Mild OSA**	**Moderate to severe OSA**
N	12,342	3,225	3,844	1,158	2,121	1,909	7,563	2,122	1,270
Gender N (%)									
Female	7,244 (50.9)	1,941 (51.0)	2,581 (60.0)	646 (48.4)	1,340 (55.1)	1,102 (50.3)	4,816 (55.4)	1,117 (42.4)	508 (31.9)
Male	5,098 (49.1)	1,284 (49.0)	1,263 (40.0)	512 (51.6)	781 (44.9)	807 (49.7)	2,747 (44.6)	1,005 (57.6)	762 (68.1)
Age									
Mean (SD)	41.51 (15.05)	37.58 (13.49)	44.70 (14.53)	43.46 (14.80)	37.94 (15.98)	43.03 (15.07)	37.87 (14.03)	50.46 (12.83)	52.62 (12.91)
BMI									
Mean (SD)	29.40 (6.13)	28.06 (5.19)	30.21 (6.70)	30.49 (6.28)	28.75 (6.35)	30.28 (6.70)	28.30 (5.66)	31.47 (6.05)	33.71 (6.25)
DM status at visit 1 N (%)									
Normoglycemic	5,090 (48.4)	1,651 (57.5)	1,387 (43.4)	416 (42.4)	959 (54.6)	722 (44.3)	3,742 (56.8)	544 (28.8)	216 (18.3)
Hyperglycemic	4,839 (36.6)	1,161 (33.8)	1,542 (37.2)	473 (41.2)	722 (30.2)	740 (38.2)	2,723 (33.1)	996 (46.8)	598 (47.1)
Diabetic	2,413 (15.0)	413 (8.8)	915 (19.4)	269 (16.4)	440 (15.2)	447 (17.4)	1,098 (10.1)	582 (24.4)	456 (34.6)
DM status at visit 2 N (%)									
Normoglycemic	2,462 (22.5)	826 (27.5)	679 (18.9)	203 (18.8)	434 (24.3)	344 (21.6)	1,854 (26.9)	258 (13.6)	98 (7.5)
Hyperglycemic	3,891 (28.6)	1,023 (28.1)	1,232 (30.4)	368 (30.2)	567 (23.3)	631 (32.2)	2,364 (27.4)	757 (34.8)	392 (31.5)
Diabetic	2,438 (14.7)	451 (9.6)	891 (17.7)	254 (16.2)	403 (13.4)	462 (18.1)	1,160 (10.5)	584 (24.0)	445 (31.0)
Missing DM status	3,551 (34.2)	925 (34.8)	1,042 (33.0)	333 (34.8)	717 (39.0)	472 (28.1)	2,185 (35.2)	523 (27.6)	335 (30.0)

Bold font represent column names.

Supplementary Table 4: Characteristics of MGB dataset stratified by T2D status.

**Characteristic**	**No T2D**	**T2D**	**Overall**
N	33,489	2,934	36,423
Gender N (%)			
Female	19,022 (56.8%)	1,325 (45.2%)	20,347 (55.9%)
Male	14,467 (43.2%)	1,609 (54.8%)	16,076 (44.1%)
Age			
Mean (SD)	57.7 (17.0)	68.6 (12.6)	58.6 (17.0)
Median [Min, Max]	60.0 [21.0, 104]	70.0 [25.0, 101]	61.0 [21.0, 104]
Self-reported background			
White	28,252 (84.4%)	2,120 (72.3%)	30,372 (83.4%)
Black	1,473 (4.4%)	354 (12.1%)	1,827 (5.0%)
Hispanic	1,099 (3.3%)	202 (6.9%)	1,301 (3.6%)
Asian	692 (2.1%)	61 (2.1%)	753 (2.1%)
American Indian/Alaskan Native	42 (0.1%)	8 (0.3%)	50 (0.1%)
More than one	309 (0.9%)	21 (0.7%)	330 (0.9%)
Unknown	1,622 (4.8%)	168 (5.7%)	1,790 (4.9%)
BMI			
Mean (SD)	28.2 (8.01)	32.8 (7.32)	28.6 (8.05)
Median [Min, Max]	27.1 [2.66, 748]	31.9 [11.7, 173]	27.5 [2.66, 748]
Missing	3,614 (10.8%)	182 (6.2%)	3,796 (10.4%)

Bold font represent column names.

Supplementary Table 5: Characteristics of HCHS/SOL target population stratified by self-reported Hispanic background.

**Characteristic**	**Central American**	**Cuban**	**Dominican**	**Mexican**	**Puerto Rican**	**South American**	**More than one/Other heritage**	**Overall**
N	1,295	2,009	1,177	4,502	2,149	803	382	12,317
Gender N (%)								
Female	767 (59.2%)	1,053 (52.4%)	772 (65.6%)	2,715 (60.3%)	1,246 (58.0%)	471 (58.7%)	203 (53.1%)	7,227 (58.7%)
Male	528 (40.8%)	956 (47.6%)	405 (34.4%)	1,787 (39.7%)	903 (42.0%)	332 (41.3%)	179 (46.9%)	5,090 (41.3%)
Age								
Mean (SD)	44.8 (13.4)	49.3 (13.0)	45.6 (14.3)	44.6 (13.8)	48.2 (14.0)	47.0 (13.2)	39.6 (15.2)	46.1 (13.9)
BMI								
Mean (SD)	29.9 (5.86)	29.2 (5.82)	29.4 (5.76)	29.8 (5.91)	30.9 (6.89)	28.7 (5.21)	30.0 (6.46)	29.8 (6.06)
Missing N (%)	2 (0.2%)	4 (0.2%)	3 (0.3%)	12 (0.3%)	10 (0.5%)	2 (0.2%)	0 (0%)	33 (0.3%)
DM status at baseline N (%)								
Diabetic	223 (17.2%)	352 (17.5%)	212 (18.0%)	910 (20.2%)	557 (25.9%)	104 (13.0%)	48 (12.6%)	2,406 (19.5%)
Hyperglycemic	516 (39.8%)	846 (42.1%)	438 (37.2%)	1,770 (39.3%)	802 (37.3%)	323 (40.2%)	133 (34.8%)	4,828 (39.2%)
Normoglycemic	556 (42.9%)	811 (40.4%)	527 (44.8%)	1,822 (40.5%)	790 (36.8%)	376 (46.8%)	201 (52.6%)	5,083 (41.3%)
Incident DM N (%)								
No incident	252 (19.5%)	398 (19.8%)	255 (21.7%)	903 (20.1%)	378 (17.6%)	182 (22.7%)	91 (23.8%)	2,459 (20.0%)
Incident	646 (49.9%)	1,010 (50.3%)	575 (48.9%)	2,481 (55.1%)	1,062 (49.4%)	410 (51.1%)	137 (35.9%)	6,321 (51.3%)
Missing	397 (30.7%)	601 (29.9%)	347 (29.5%)	1,118 (24.8%)	709 (33.0%)	211 (26.3%)	154 (40.3%)	3,537 (28.7%)

Bold font represent column names.

Supplementary Table 6: Associations of T2D PRS with OSA in analyses adjusted for WHR instead of BMI

**Outcome**	**n**	**PRS**	**OR**	**95% CI**	**Obesity measure**
Moderate to severe OSA	10925	PRSsum	1.17	[1.01; 1.35]	BMI
Moderate to severe OSA	9060	gapPRSsum	1.15	[1.00; 1.32]	BMI
Moderate to severe OSA	10925	mgbPRSsum	1.17	[1.04; 1.32]	BMI
Mild to severe OSA	10925	PRSsum	1.20	[1.08;1.33]	BMI
Mild to severe OSA	9060	gapPRSsum	1.14	[1.03;1.27]	BMI
Mild to severe OSA	10925	mgbPRSsum	1.15	[1.06;1.26]	BMI
Moderate to severe OSA	10924	PRSsum	1.10	[0.96;1.25]	WHR
Moderate to severe OSA	9062	gapPRSsum	1.06	[0.96;1.20]	WHR
Moderate to severe OSA	10924	mgbPRSsum	1.12	[0.99;1.25]	WHR
Mild to severe OSA	10924	PRSsum	1.17	[1.06;1.29]	WHR
Mild to severe OSA	9062	gapPRSsum	1.10	[1.00;1.21]	WHR
Mild to severe OSA	10924	mgbPRSsum	1.13	[1.04;1.23]	WHR

Estimated associations of T2D PRSs with OSA outcomes (mild-to-severe OSA versus no OSA, and moderate-to-severe OSA versus no and mild OSA). Associations were estimated in survey logistic regression adjusted for age, sex, the 5 first genetic principal components, and either BMI or WHR. Bold font represent column names.
T2D: type 2 diabetes; OSA: obstructive sleep apnea; BMI: body mass index; OR: odds ratios; WHR: waist-to-hip ratio; CI: confidence interval.

Supplementary Table 7: Estimated associations between T2D PRSs and moderate-to-severe OSA when adjusting for potential confounders of the T2D-OSA association

**Outcome**	**Confounder**	**N case**	**N control**	**PRS**	**OR**	**95% CI**
Moderate to severe OSA	Education level	1153	8727	PRSsum	1.17	[1.00;1.35]
Moderate to severe OSA	Medication: Statin	1133	8564	PRSsum	1.16	[0.99;1.35]
Moderate to severe OSA	Income range	1153	8727	PRSsum	1.17	[1.00;1.36]
Moderate to severe OSA	Vigorous Physical Activity	1267	9620	PRSsum	1.17	[1.00;1.35]
Moderate to severe OSA	Total Physical Activity	1267	9621	PRSsum	1.17	[1.01;1.35]
Moderate to severe OSA	Background	1269	9656	PRSsum	1.17	[1.00;1.34]
Moderate to severe OSA	Education level	996	7211	gapPRSsum	1.15	[0.99;1.33]
Moderate to severe OSA	Medication: Statin	980	7077	gapPRSsum	1.15	[0.99;1.33]
Moderate to severe OSA	Income range	996	7211	gapPRSsum	1.16	[1.00;1.33]
Moderate to severe OSA	Vigorous Physical Activity	1097	7933	gapPRSsum	1.15	[1.00;1.31]
Moderate to severe OSA	Total Physical Activity	1097	7934	gapPRSsum	1.15	[1.00;1.31]
Moderate to severe OSA	Background	1097	7963	gapPRSsum	1.15	[1.00;1.31]
Moderate to severe OSA	Education level	1153	8727	mgbPRSsum	1.19	[1.05;1.35]
Moderate to severe OSA	Medication: Statin	1133	8564	mgbPRSsum	1.19	[1.04;1.34]
Moderate to severe OSA	Income range	1153	8727	mgbPRSsum	1.19	[1.05;1.35]
Moderate to severe OSA	Vigorous Physical Activity	1267	9620	mgbPRSsum	1.17	[1.03;1.32]
Moderate to severe OSA	Total Physical Activity	1267	9621	mgbPRSsum	1.17	[1.03;1.32]
Moderate to severe OSA	Background	1269	9656	mgbPRSsum	1.18	[1.04;1.32]

Estimated associations of T2D PRSs with moderate-to-severe OSA versus no and mild OSA. Associations were estimated in survey logistic regression adjusted for age, sex, the 5 first genetic principal components, BMI, and each potential confounder. Bold font represent column names.

T2D: type 2 diabetes; OSA: obstructive sleep apnea; BMI: body mass index; OR: odds ratios; WHR: waist-to-hip ratio; CI: confidence interval.

Supplementary Table 8: Estimated associations between T2D PRSs and mild-to-severe OSA when adjusting for potential confounders of the T2D-OSA association

**Outcome**	**Confounder**	**N case**	**N control**	**PRS**	**OR**	**95% CI**
Mild to severe OSA	Education level	3025	6855	PRSsum	1.19	[1.06;1.33]
Mild to severe OSA	Medication: Statin	2971	6726	PRSsum	1.19	[1.05;1.32]
Mild to severe OSA	Income range	3025	6855	PRSsum	1.20	[1.06;1.33]
Mild to severe OSA	Vigorous Physical Activity	3338	7549	PRSsum	1.20	[1.07;1.33]
Mild to severe OSA	Total Physical Activity	3338	7550	PRSsum	1.20	[1.08;1.33]
Mild to severe OSA	Background	3344	7581	PRSsum	1.20	[1.07;1.33]
Mild to severe OSA	Education level	2615	5592	gapPRSsum	1.13	[1.01;1.25]
Mild to severe OSA	Medication: Statin	2570	5487	gapPRSsum	1.12	[1.00;1.25]
Mild to severe OSA	Income range	2615	5592	gapPRSsum	1.13	[1.01;1.26]
Mild to severe OSA	Vigorous Physical Activity	2885	6145	gapPRSsum	1.14	[1.03;1.26]
Mild to severe OSA	Total Physical Activity	2885	6146	gapPRSsum	1.14	[1.03;1.26]
Mild to severe OSA	Background	2888	6172	gapPRSsum	1.15	[1.03;1.26]
Mild to severe OSA	Education level	3025	6855	mgbPRSsum	1.16	[1.05;1.27]
Mild to severe OSA	Medication: Statin	2971	6726	mgbPRSsum	1.15	[1.04;1.26]
Mild to severe OSA	Income range	3025	6855	mgbPRSsum	1.16	[1.05;1.27]
Mild to severe OSA	Vigorous Physical Activity	3338	7549	mgbPRSsum	1.15	[1.05;1.25]
Mild to severe OSA	Total Physical Activity	3338	7550	mgbPRSsum	1.15	[1.05;1.26]
Mild to severe OSA	Background	3344	7581	mgbPRSsum	1.16	[1.05;1.26]

Estimated associations of T2D PRSs with mild-to-severe OSA versus no OSA. Associations were estimated in survey logistic regression adjusted for age, sex, the 5 first genetic principal components, BMI, and each potential confounder. Bold font represent column names.

T2D: type 2 diabetes; OSA: obstructive sleep apnea; BMI: body mass index; OR: odds ratios; WHR: waist-to-hip ratio; CI: confidence interval.

Supplementary Table 9: Results from bidirectional multivariate MR (MVMR) analysis of T2D and OSA adjusted for BMI

**Threshold**	**Exposure**	**Outcome**	**N SNPs**	**OR**	**95% CI**
5×10^{-8}	T2D	OSA	192	0.965	[0.94;0.98]
5×10^{-7}	T2D	OSA	213	0.967	[0.95;0.98]
5×10^{-5}	T2D	OSA	215	0.969	[0.95;0.98]
5×10^{-8}	OSA	T2D	10	2.006	[1.84;2.17]
5×10^{-7}	OSA	T2D	11	1.976	[1.82;2.13]
5×10^{-5}	OSA	T2D	11	1.727	[1.62;1.83]

BMI was used as an additional exposure in all analyses. Results are not reported for BMI because they do not reflect the causal effect of BMI on the outcome, given conditioning on a collider. Threshold is the p-value threshold used for variant selection to serve as instrumental variables. N SNPs is the number of IVs used. Bold font represent column names.

T2D: type 2 diabetes; OSA: obstructive sleep apnea; BMI: body mass index; OR: odds ratios; CI: confidence interval; SNP: single nucleotide polymorphism.

Supplementary References

1. Yu, S., et al., *Toward high-throughput phenotyping: unbiased automated feature extraction and selection from knowledge sources.* J Am Med Inform Assoc, 2015. **22**(5): p. 993-1000.
2. Mahajan, A., et al., *Multi-ancestry genetic study of type 2 diabetes highlights the power of diverse populations for discovery and translation.* Nat Genet, 2022. **54**(5): p. 560-572.
3. Vujkovic, M., et al., *Discovery of 318 new risk loci for type 2 diabetes and related vascular outcomes among 1.4 million participants in a multi-ancestry meta-analysis.* Nat Genet, 2020. **52**(7): p. 680-691.
