# Supplementary material for: Obstructive sleep apnea mediates genetic risk of Diabetes Mellitus in Hispanic and Latino communities
Source: Commun Med (Lond). 2025 Sep 25;5:398. doi: 10.1038/s43856-025-01107-y (PMC12462445; doi:10.1038/s43856-025-01107-y)
Supplement: Supplementary file 3 — Description of Additional Supplementary Files [file 43856_2025_1107_MOESM3_ESM.pdf]

Description of Additional Supplementary files

File name: Supplementary Data 1

Description: complete lists of SNPs used in estimation of causal effect of T2D on OSA and of OSA on T2D.

File name: Supplementary Data 2

Description: The SNP-level data for the MVMR analysis.

File name: Supplementary Data 3

Description: The source data for Figures 1 and 2.
