# Supplementary material for: Obstructive sleep apnea mediates genetic risk of Diabetes Mellitus in Hispanic and Latino communities
Source: Commun Med (Lond). 2025 Sep 25;5:398. doi: 10.1038/s43856-025-01107-y (PMC12462445; doi:10.1038/s43856-025-01107-y)
Supplement: Supplementary file 7 — Reporting summary [file 43856_2025_1107_MOESM7_ESM.pdf]

Corresponding author(s): Tamar Sofer

Last updated by author(s): Jul 4, 2025

Reporting Summary

Nature Portfolio wishes to improve the reproducibility of the work that we publish. This form provides structure for consistency and transparency in reporting. For further information on Nature Portfolio policies, see our Editorial Policies and the Editorial Policy Checklist.

Statistics

For all statistical analyses, confirm that the following items are present in the figure legend, table legend, main text, or Methods section.

n/a	Confirmed	
☐	☒	The exact sample size (*n*) for each experimental group/condition, given as a discrete number and unit of measurement
☐	☒	A statement on whether measurements were taken from distinct samples or whether the same sample was measured repeatedly
☐	☒	The statistical test(s) used AND whether they are one- or two-sided<br>*Only common tests should be described solely by name; describe more complex techniques in the Methods section.*
☐	☒	A description of all covariates tested
☒	☐	A description of any assumptions or corrections, such as tests of normality and adjustment for multiple comparisons
☐	☒	A full description of the statistical parameters including central tendency (e.g. means) or other basic estimates (e.g. regression coefficient) AND variation (e.g. standard deviation) or associated estimates of uncertainty (e.g. confidence intervals)
☐	☒	For null hypothesis testing, the test statistic (e.g. *F*, *t*, *r*) with confidence intervals, effect sizes, degrees of freedom and *P* value noted<br>*Give P values as exact values whenever suitable.*
☒	☐	For Bayesian analysis, information on the choice of priors and Markov chain Monte Carlo settings
☒	☐	For hierarchical and complex designs, identification of the appropriate level for tests and full reporting of outcomes
☐	☒	Estimates of effect sizes (e.g. Cohen's *d*, Pearson's *r*), indicating how they were calculated

Our web collection on statistics for biologists contains articles on many of the points above.

Software and code

Policy information about availability of computer code

Data collection	no software was used
Data analysis	PLINK (v2.0), PRS-CSx, PRSice, GWAMA , ADMIXTURE, PC-AiR, PC-Relate, GENESIS, mediation (v4.5.0), TwoSampleMR (v0.5.11), MR-PRESSO, MR-RAPS

For manuscripts utilizing custom algorithms or software that are central to the research but not yet described in published literature, software must be made available to editors and reviewers. We strongly encourage code deposition in a community repository (e.g. GitHub). See the Nature Portfolio guidelines for submitting code & software for further information.

Data

Policy information about availability of data

All manuscripts must include a data availability statement. This statement should provide the following information, where applicable:

- Accession codes, unique identifiers, or web links for publicly available datasets
- A description of any restrictions on data availability
- For clinical datasets or third party data, please ensure that the statement adheres to our policy

Ancestry-specific summary statistics from GWAS of DM published in 2022 (Mahajan et al) in the DIAGRAM consortium were downloaded from https://diagram-consortium.org/downloads.html. HARE-group specific summary statistics from GWAS of DM in MVP published in 2020 (Vujkovic et al) were downloaded by dbGaP application to study accession phs001672. SNPs and weights for ancestry-specific T2D-PRSs are provided in the GitHub repository https://github.com/

YanaHrytsenko/DM_PRS_OSA_mediation. HCHS/SOL data are available through application to the data base of genotypes and phenotypes (dbGaP) accession phs000810, or via a data use agreement with the HCHS/SOL Data Coordinating Center (DCC) at the University of North Carolina at Chapel Hill, see collaborators website: https://sites.cscc.unc.edu/hchs/.

Human research participants

Policy information about studies involving human research participants and Sex and Gender in Research.

Reporting on sex and gender	Findings of this study apply to both, sex and gender. Quality control was performed, including checks that biological sex (based on genetic data) matched reported gender.
Population characteristics	Of the HCHS/SOL target population, 50.9% were females and the mean age and BMI were 41.51 (SD = 15.05) and 29.4 (SD = 6.13) respectively. At visit 1, 48.4% of the target population was normoglycemic, 36.6% was hyperglycemic and 15% met criteria for diabetes. Focusing on the population who did not have DM (normal or hyperglycemic) at visit 1 and participated in visit 2, 8.08% of the HCHS/SOL target population had incident DM at visit 2.
Recruitment	Individuals were recruited to the study via a multi-stage sampling design. The study enrolled 16,415 adult participants (18- to 74-year-old at baseline) from four geographic areas: Bronx, NY, Chicago, IL, Miami, FL, and San Diego, CA, with enrollment between 2008-2011 (visit 1). Individuals self-identified with Hispanic/Latino backgrounds including Cuban, Central American, Dominican, Mexican, Puerto Rican, and South American. During the baseline exam (visit 1), individuals responded to various questionnaires, including sleep-related, and health measures including anthropometry, scanned medications, and fasting blood samples, were collected. HCHS/SOL participants were invited to participate in a second visit (visit 2; N = 11,623), which took place from 2014-2017, on average 6 years following visit 1.
Ethics oversight	The HCHS/SOL was approved by the institutional review boards (IRBs) at each field center, where all participants gave written informed consent, and by the Non-Biomedical IRB at the University of North Carolina at Chapel Hill, to the HCHS/SOL Data Coordinating Center. All IRBs approving the HCHS/SOL study are: Non-Biomedical IRB at the University of North Carolina at Chapel Hill. Chapel Hill, NC; Einstein IRB at the Albert Einstein College of Medicine of Yeshiva University. Bronx, NY; IRB at Office for the Protection of Research Subjects (OPRS), University of Illinois at Chicago. Chicago, IL; Human Subject Research Office, University of Miami. Miami, FL; Institutional Review Board of San Diego State University, San Diego, CA. All methods and analyses of HCHS/ SOL participants' materials and data were carried out in accordance with human subject research guidelines and regulations. This work was approved by the Mass General Brigham IRB and by the Beth Israel Deaconess Medical Center Committee on Clinical Investigations.

Note that full information on the approval of the study protocol must also be provided in the manuscript.

Field-specific reporting

Please select the one below that is the best fit for your research. If you are not sure, read the appropriate sections before making your selection.

☒ Life sciences ☐ Behavioural & social sciences ☐ Ecological, evolutionary & environmental sciences

For a reference copy of the document with all sections, see nature.com/documents/nr-reporting-summary-flat.pdf

Life sciences study design

All studies must disclose on these points even when the disclosure is negative.

Sample size	Sample size was reported. We used all available observations.
Data exclusions	No data were excluded.
Replication	We did not perform replication. We developed polygenic scores using multiple independent datasets (GWAS summary statistics, and MGB biobank), and applied them in the HCHS/SOL.
Randomization	Randomization was not relevant to this study.
Blinding	Blinding was not relevant to this study.

Reporting for specific materials, systems and methods

We require information from authors about some types of materials, experimental systems and methods used in many studies. Here, indicate whether each material, system or method listed is relevant to your study. If you are not sure if a list item applies to your research, read the appropriate section before selecting a response.

Materials & experimental systems

n/a	Involved in the study
☒	☐ Antibodies
☒	☐ Eukaryotic cell lines
☒	☐ Palaeontology and archaeology
☒	☐ Animals and other organisms
☒	☐ Clinical data
☒	☐ Dual use research of concern

Methods

n/a	Involved in the study
☒	☐ ChIP-seq
☒	☐ Flow cytometry
☒	☐ MRI-based neuroimaging
